# Early-Onset Colorectal Cancer: Prevalence, Risk Factors, and Clinical Features Among Commercially Insured Adults in the United States

**DOI:** 10.7759/cureus.49432

**Published:** 2023-11-26

**Authors:** Christopher Tait, Ankoor H Patel, Alexander Chen, You Li, Carlos D Minacapelli, Vinod Rustgi

**Affiliations:** 1 Department of Medicine, Division of Gastroenterology and Hepatology, Rutgers Robert Wood Johnson Medical School, New Brunswick, USA; 2 Department of Internal Medicine, Rutgers Robert Wood Johnson Medical School, New Brunswick, USA

**Keywords:** young onset colorectal cancer, early-onset colon cancer, early rectal cancer, colon cancer survillence, early-onset colorectal cancer

## Abstract

Background: The incidence of colorectal cancer (CRC) in patients younger than 50 has been rising over the last several decades, accounting for up to 25% of total cases. Despite the screening age recently being lowered to 45, a significant proportion of cases would still arise at younger ages prior to screening. Nonfamilial early-onset CRC remains a particular concern. Identification of risk factors and clinical features in this age group is needed to improve detection.

Methods: In this retrospective cohort analysis using claims data from the Truven Health MarketScan® Commercial Claims insurance database from 2007 to 2017, patients were identified with colon and rectal cancer, compared across three age groups (ages 18-40, 40-50, and >50), and analyzed for risk factors and clinical features.

Results: Female sex was more prevalent in the younger age group compared to age >50 (54% and 51.9% vs. 49.6%), with little change noted between rectal cancer age groups by sex. A higher percentage of younger patients were in the obese age groups compared with older groups for colon cancer, particularly the morbidly obese with BMI >40 (24.94%, 25.75%, and 21.34% in the three age groups). Abdominal pain was a common presenting symptom identified in the age groups <50 compared with age >50 (25% and 19% vs. 14%), along with hematochezia, weight loss, and anemia.

Conclusions: Morbid obesity and female sex may be important risk factors among patients with early-onset CRC. The presence of abdominal pain was more common among the early-onset CRC cohort.

## Introduction

Young adults with colorectal cancer (CRC) represent an emerging epidemic among cancer patients in the United States over the last three decades. CRC is the third most common cancer worldwide and the second leading cause of cancer mortality in both genders [1]. The incidence of CRC across all age groups in high-income countries has been stable or decreased in the past several decades due to widespread screening programs and changing risk factors, including decreased smoking [1]. Screening tests and preventive management strategies for CRC in patients older than 50 have resulted in incidence rates that have decreased by 2-3% per year in both men and women since 2000 in the United States [2]. While detection and prevention of CRC in this age group represents a major public health milestone, an increasing number of cases in patients age <50, which are termed early-onset CRC, is forcing physicians and epidemiologists to refocus management on this younger demographic [3]. Additionally, there are increasing reports of patients diagnosed with very early-onset CRC, seen in the age group of adults age <40, including in many patients with no known genetic or familial predisposition [4].

The median age of patients with colon cancer and rectal cancer is 70 years and 63 years, respectively [5,6]. Among all US adults with colon and rectal cancer in 2010, cases in patients younger than age 50 accounted for 4.8% and 9.5% of colon and rectal cancer cases, respectively [7,8]. Epidemiological studies indicate that since 1994, the incidence rate of CRC in patients age <55 has increased by 2% annually, with the highest increase in rectosigmoid cancers between the ages of 18 and 35 [7]. Current modeling of these trends predicts the proportional incidence of both cancers will double to an estimated 11% and 22% for colon and rectal cancer, respectively, by 2030 in this younger age demographic. While certain risk factors help identify patients at high risk for early-onset CRC, over 50% of early-onset cases are sporadic [9]. Additionally, previous series have not gone into detail regarding the features of very early-onset adults with CRC in the age group 18-40, leaving risk factors in this demographic unaccounted for in screening models outside of the previously mentioned inflammatory bowel disease, genetic syndromes, or certain features of family history.

A deeper understanding of the demographics and risk factors associated with gastrointestinal cancers in younger patient populations can help improve screening practices. It can also provide impetus to interventions aimed at reversing potentially modifiable risk factors. Several recent reviews have addressed the epidemic of early-onset cancers and identified multiple risk factors, including obesity during adolescence and early adulthood, sedentary lifestyle, metabolic syndrome, alcohol use, and diet-related factors [10,11]. Previous series looking into younger age groups have mostly looked into risk factors for patients age <50, but the identification of specific risk features in the very early-onset group (age <40) remains to be studied and is one of the major focuses of this analysis.

Further analysis at a large scale is needed to identify demographic groups, clinical features, and risk factors that may help further guide screening guidelines. Consequently, we sought to evaluate demographic, clinical features, and comorbidity risk factors associated with colon and rectal cancers in younger patient populations, including stratification into early-onset (age 40 to 50) and very early-onset (age 18 to 40) groups based on a database analysis of insurance claims (Truven Health MarketScan® Commercial Claims (MSCC)).

This article was previously posted on a preprint server.

## Materials and methods

Data source

The Truven Health MSCC database from January 1, 2007, to December 31, 2017, was accessed to extract retrospective claims-based healthcare data. The claims data represent healthcare records from government and public organizations, large employers, and health plans from approximately 350 payers annually. The MSCC database includes longitudinal, individual-level data for health insurance claims including inpatient, outpatient, and prescription drugs. The MSCC database contains de-identified data that is compliant with all United States patient confidentiality requirements, including the Health Insurance Portability and Accountability Act of 1996. The Internal Review Board (IRB) of Rutgers Robert Wood Johnson Medical School approved the protocol of this study.

Study sample and variables

**Figure 1:**
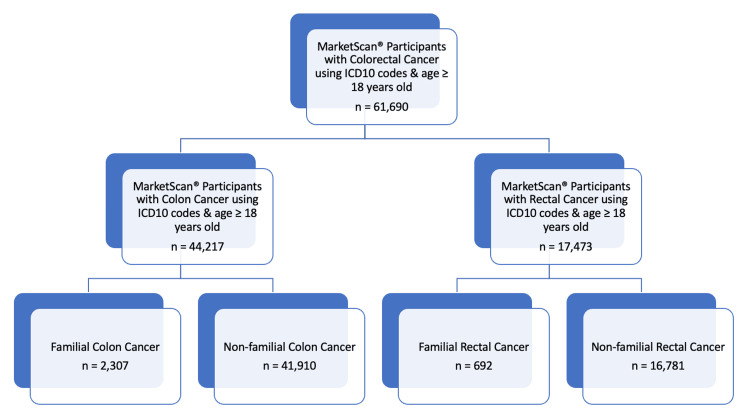
Diagram for study sample selection

The study sample included 18- to 65-year-old MSCC enrollees with at least one documented inpatient admission or outpatient service diagnosis for CRC. Accordingly, between January 1, 2007, and December 31, 2017, CRC was defined according to the International Statistical Classification of Diseases and Related Health Problems (ICD), 10th Revision, Clinical Modification (ICD-10-CM) codes [12]. The cohort breakdown is summarized in Figure 1.

We defined the age group ≤40 as the younger or very early-onset group, 40-50 as the early-onset age group, and ≥50 to 65 as the older age group. Symptoms such as hematochezia, anemia, dysphagia, abdominal pain, and weight loss were captured from records at any point prior to index (and including index date) by ICD codes. In addition, history of smoking, alcohol use, and BMI information were captured prior to the index by ICD coding. Statin use and death information were captured before and after the index date.

The date of the first CRC diagnosis was defined as the index date for all study participants. Baseline demographics, including age, gender, region of residence, and the type of health insurance plan, were obtained from the index date records. A comorbidity profile was measured for each age group during the baseline period using ICD-10 codes acquired from inpatient admissions and outpatient services. The profile included participants’ statuses on Crohn's disease (CD), ulcerative colitis (UC), polyposis syndromes, including Peutz-Jeghers (PJ), benign neoplasm of the colon, including juvenile polyposis (JP), family history of colonic polyps, including familial adenomatous polyposis (FAP), and GI hemorrhage, including GI bleeding. Lynch syndrome status could not be extrapolated reliably from the database based on search parameters, so it was not included. Familial vs. non-familial cancer status was extrapolated based on coding history for family history of CRC and personal or family history of JP syndrome, PJ syndrome, FAP, or family history of GI cancer. In addition, a total weighted Charlson Comorbidity Index (CCI) score was estimated for each age group during the baseline period using algorithms provided by Quan et al. [13].

Control group patients

An available claims-based control group consisting of patients with chronic liver disease (CLD) with one primary or secondary ICD-9/ICD-10 code (during the period of 2015-2017) for hepatocellular carcinoma, compensated cirrhosis, hepatitis C virus, chronic hepatitis B virus, hepatitis D virus, alcoholic fatty liver, nonalcoholic fatty liver disease (NAFLD), hepatic encephalopathy, autoimmune hepatitis, hepatitis E virus, primary biliary cirrhosis or primary sclerosing cholangitis, and malignant neoplasm of intrahepatic bile ducts. This was an established, well-studied cohort used in previous analyses and analyzed for differences in risk factors [14].

Statistical analysis

We compared CRC baseline characteristics and comorbidity profiles for those under the specific age groups 18-40, 40-50, and 50-65 and classified ≥50 as the control group. We analyzed the cancer patients in each group and examined outcomes compared with control patients. We also performed subgroup analysis based on the history of familial/hereditary GI cancer, which included verifiable heritable GI genetic cancer syndromes and a family history of GI cancer. Wald Chi-square tests were performed to test the associations between age ≤40 and 40-50 and age ≥50 and reported as p-values, with a significance threshold defined as <0.05.

## Results

Colon cancer

A total of 61,690 patients were identified based on ICD-10 codes for CRC with patients identified by age group (Figure 1). Among these patients, 44,217 were identified with colon cancer. The demographics, comorbidities, and clinical features of these patients by age group with colon cancer in comparison with control group patients are described in Table 1. The majority of patients diagnosed with colon cancer were in the non-familial group (94.78%) and were above age 50 (75.97%). Among all patients with colon cancer, 7.58% were diagnosed before the age of 40 years, 16.45% of patients were diagnosed between 40 and 50 years, and 75.97% were diagnosed after the age of 50 years. A subgroup analysis excluding patients with hereditary syndromes and a family history of GI cancer was performed (Table 2), with 7.58% of cases age <40, 16.45% of age 40-50, and 75.97% of age >50. All p-values reported between age groups reached significance.

**Table 1:** Colon cancer patients by age with controls ‡ estimated during the 12‐month baseline period

Patient characteristics	Cancer age ≤40	Control age ≤40	Cancer age 40-50	Control age 40-50	Cancer age ≥50	Control age >50	Age ≤40 vs. ≥50	Age 40-50 vs. ≥50
	n=3,346 (7.57%)	(n=179,828)	n=7,257 (16.41%)	n=145,512	n=33,614 (76.02%)	n=326,697	p-value	p-value
Age (mean (SD))	33.95 (5.42)	31.45 (6.23)	45.78 (2.48)	45.25 (2.54)	57.66 (4.27)	57.02 (4.22)	<0.0001	<0.0001
Age group (n (%))							<0.0001	<0.0001
18-30	787 (23.52)	72,064 (40.07)	0	0	0	0		
30-40	2,559 (76.48)	107,764 (59.93)	0	0	0	0		
40-50	0	0	7,257 (100.00)	142,512 (100.00)	0	0		
50+	0	0	0	0	33,614 (100.00)	326,697 (100.00)		
Gender (n (%))							<0.0001	0.0004
Male	1,537 (45.94)	83,743 (46.57)	3,485 (48.02)	69,755 (48.95)	16,920 (50.34)	153,545 (47.00)		
Female	1,809 (54.06)	96,085 (53.43)	3,772 (51.98)	72,757 (51.05)	16,694 (49.66)	173,152 (53.00)		
Year (n (%))							<0.0001	0.1019
2015	953 (28.48)	55,796 (31.03)	2,432 (33.51)	44,700 (31.37)	11,393 (33.89)	104,435 (31.97)		
2016	1,299 (38.82)	62,922 (34.99)	2,882 (39.71)	49,379 (34.65)	13,624 (40.53)	112,873 (43.55)		
2017	1,094 (32.70)	61,110 (33.98)	1,943 (26.77)	48,433 (33.99)	8,597 (25.58)	109,389 (33.48)		
Region of residence (n (%))							<0.0001	<0.0001
Northeast	623 (18.62)	31,820 (17.69)	1,227 (16.91)	25,132 (17.64)	6,462 (19.22)	62,338 (39.08)		
Northcentral	590 (17.63)	31,491 (17.51)	1,353 (18.64)	24,061 (16.88)	6,698 (19.93)	58,772 (17.99)		
South	1,619 (48.39)	87,194 (48.49)	3,598 (49.58)	72,117 (50.60)	16,170 (48.10)	159,386 (48.79)		
West	499 (14.91)	28,947 (16.10)	1,052 (14.50)	20,872 (14.65)	4,188 (12.46)	45,287 (13.86)		
Unknown	15 (0.45)	376 (0.21)	27 (0.37)	330 (0.23)	96 (0.29)	914 (0.28)		
Charlson comorbidity index ‡ (mean (SD))	1.59 (0.82)	0.37 (0.67)	1.73 (0.91)	0.61 (0.90)	2.00 (1.18)	1.02 (1.23)	<0.0001	<0.0001
Charlson comorbidity index (n (%))							<0.0001	<0.0001
0	0	128,096 (71.23)	0	83,186 (58.37)	0	141,664 (43.36)		
1	1,899 (56.75)	40,608 (22.58)	3,578 (49.30)	40,458 (28.39)	14,116 (41.99)	98,533 (30.16)		
2	1,062 (31.74)	8,522 (4.74)	2,547 (35.10)	12,999 (9.12)	11,112 (33.06)	49,041 (15.01)		
3	279 (8.34)	1,903 (1.06)	810 (11.16)	3,961 (2.78)	4,962 (14.76)	21,860 (6.69)		
4+	106 (3.17)	699 (0.39)	322 (4.44)	1,908 (1.34)	3,424 (10.19)	15,599 (4.77)		
BMI (n (%))							0.0237	0.0254
≤25	83 (2.48)	3,011 (1.67)	173 (2.34)	1,645 (1.13)	814 (2.42)	4,795 (1.47)		
25-30	100 (2.99)	4,265 (2.37)	255 (3.51)	4,362 (3.68)	1,256 (3.73)	11,972 (3.66)		
30-35	83 (2.48)	5,437 (2.97)	239 (3.29)	5,617 (3.86)	1,138 (3.38)	13,197 (4.04)		
35-40	62 (1.85)	4,174 (2.32)	129 (1.78)	3,979 (2.73)	555 (1.65)	7,903 (2.42)		
40+	109 (3.26)	11,936 (6.64)	276 (3.80)	9,079 (6.24)	1,021 (3.03)	14,462 (4.43)		
Family history of GI malignancies (n (%))	360 (10.76)	2,253 (1.25)	601 (8.28)	4,218 (2.96)	2,077 (6.18)	12,087 (3.70)	<0.0001	<0.0001
Personal history of other non-GI malignancies (n (%))	373 (11.15)	664 (0.37)	954 (13.15)	1,625 (1.14)	4,771 (14.19)	8,513 (2.61)	<0.0001	0.0197
Peutz-Jeghers (n (%))	0	26 (0.01)	1 (0.01)	20 (0.01)	3 (0.01)	32 (0.01)	0.5847	0.7046
Juvenile polyposis (n (%))	126 (3.77)	284 (0.16)	263 (3.62)	529 (0.37)	1,525 (4.54)	4,028 (1.23)	0.0395	0.0006
Familial adenomatous polyposis (n (%))	54 (1.61)	221 (0.12)	107 (1.47)	493 (0.35)	280 (0.83)	1,542 (0.47)	<0.0001	<0.0001
Crohn's colitis (n (%))	60 (1.79)	1,877 (1.04)	64 (0.88)	968 (0.68)	198 (0.59)	1,777 (0.54)	<0.0001	0.0046
Ulcerative colitis (n (%))	89 (2.66)	1,350 (0.75)	96 (1.32)	878 (0.62)	348 (1.04)	2,161 (0.66)	<0.0001	0.0321
Smoking (n (%))	454 (13.57)	26,554 (14.77)	941 (12.97)	20,638 (14.48)	5,874 (17.47)	54,890 (16.80)	<0.0001	<0.0001
Alcohol (n (%))	58 (1.73)	7,857 (4.37)	171 (2.36)	6,182 (4.34)	944 (2.81)	15,135 (4.63)	0.0003	0.0321
Statins (n (%))	11 (0.33)	1,342 (0.75)	230 (3.17)	5,025 (3.53)	2,597 (7.73)	27,354 (8.37)	<0.0001	<0.0001
Patient index symptoms								
GI bleeding (n (%))	122 (3.65)	1,161 (0.65)	290 (4.00)	1,210 (0.85)	994 (2.96)	4,150 (1.27)	0.0263	<0.0001
Hematochezia (n (%))	269 (8.04)	2,630 (1.46)	629 (8.67)	2,063 (1.45)	1,406 (4.18)	4,879 (1.49)	<0.0001	<0.0001
Anemia (n (%))	525 (15.69)	10,833 (6.02)	1,148 (15.82)	10,561 (7.41)	4,640 (13.80)	26,817 (8.21)	0.0027	<0.0001
Dysphagia (n (%))	52 (1.55)	2,046 (1.14)	113 (1.56)	2,420 (1.70)	523 (1.56)	6,767 (2.07)	0.9936	0.9939
Abdominal pain (n (%))	866 (25.88)	50,361 (28.01)	1,441 (19.86)	36,880 (25.88)	4,855 (14.44)	74,833 (22.91)	<0.0001	<0.0001
Weight loss (n (%))	111 (3.32)	1,886 (1.05)	220 (3.03)	1,480 (1.04)	945 (2.81)	5,517 (1.69)	0.0938	0.3066
Vitamin D deficiency (n (%))	198 (5.92)	13,330 (7.41)	534 (7.36)	14,531 (10.20)	2,620 (7.79)	34,992 (10.71)	<0.0001	0.2069
Death (n (%))	6 (0.18)	156 (0.09)	12 (0.17)	200 (0.14)	70 (0.21)	1,224 (0.37)	0.7246	0.459

**Table 2:** Non-familial patients with colon cancer ‡ estimated during the 12‐month baseline period

Patient characteristics	Age ≤40	Age 40-50	Age ≥50	Age ≤40 vs. ≥50	Age 40-50 vs. ≥ 50
	n=3,176 (7.58%)	n=6,896 (16.45%)	n=31,838 (75.97%)	p-value	p-value
Age (mean (SD))	33.99 (5.40)	45.78 (2.48)	57.68 (4.26)	<0.0001	<0.0001
Gender (n (%))				<0.0001	0.0018
Male	1,460 (45.97)	3,324 (48.20)	16,008 (50.28)		
Female	1,716 (54.03)	3,572 (51.80)	15,830 (49.72)		
Comorbidity profile ‡					
Charlson comorbidity index (mean (SD))	1.60 (0.82)	1.73 (0.90)	2.01 (1.18)	<0.0001	<0.0001
BMI				0.041	0.0462
≤25	78 (19.16)	159 (16.01)	767 (17.27)		
25-30	93 (22.85)	238 (23.97)	1,170 (26.35)		
30-35	78 (19.16)	225 (22.66)	1,050 (23.65)		
35-40	57 (14.00)	118 (11.88)	511 (11.51)		
40+	101 (24.82)	253 (25.48)	942 (21.22)		
Crohn's colitis (n (%))	56 (1.76)	61 (0.88)	183 (0.57)	<0.0001	0.0032
Ulcerative colitis (n (%))	80 (2.52)	83 (1.20)	308 (0.97)	<0.0001	0.0752
Personal history of other non-GI malignancies (n (%))	353 (11.11)	911 (13.21)	4,507 (14.16)	<0.0001	0.0401
Smoking (n (%))	430 (13.54)	889 (12.89)	5,491 (17.25)	<0.0001	<0.0001
Alcohol (n (%))	54 (1.70)	165 (2.39)	873 (2.74)	0.0005	0.1034
Statins (n (%))	10 (0.31)	219 (3.18)	2,433 (7.64)	<0.0001	<0.0001
Clinical features					
Hematochezia (n (%))	237 (7.46)	553 (8.02)	1,256 (3.94)	<0.0001	<0.0001
Anemia (n (%))	490 (15.43)	1,081 (15.68)	4,331 (13.60)	0.0044	<0.0001
Dysphagia (n (%))	51 (1.61)	103 (1.49)	471 (1.48)	0.575	0.9292
Abdominal pain (n (%))	796 (25.06)	1,339 (19.42)	4,524 (14.21)	<0.0001	<0.0001
Weight loss (n (%))	100 (3.15)	208 (3.02)	888 (2.79)	0.2433	0.3024
GI bleeding (n (%))	111 (3.49)	263 (3.81)	916 (2.88)	0.0491	<0.0001
Vitamin D deficiency (n (%))	181 (5.70)	495 (7.18)	2,400 (7.54)	0.0002	0.3026
Death (n (%))	6 (0.19)	12 (0.17)	69 (0.22)	0.7465	0.4815

The average age of onset for very early colon cancers was 33.99 years and 45.78 in the age 40-50 group. The majority of cases in all groups were from the South by region of residence, and this did not vary appreciably across age groups (48.39% to 49.58%). Very early-onset colon cancer patients had a significantly higher proportion of females compared to middle and older age groups (54.03% vs. 51.80% vs. 49.72%) and an increase in female proportion compared with controls in these groups. Very early-onset and early-onset colon cancer patients had a lower CCI than older colon cancer patients (CCI 4+; 3.15% vs. 4.44% vs. 10.28%; P<0.001). Compared with control patients, a higher percentage of cancer patients had two or more comorbidities noted. The majority of colon cancer patients were in the overweight and obese classes, with a slightly increased proportion in the highest BMI categories in the early-onset and middle-age groups compared with the oldest cohort (BMI >40; 24.82% and 25.48% vs. 21.22%). Compared with control group patients, the very early-onset and early-onset colon cancer groups had generally lower obesity rates than the control groups in the measured BMI categories, including in the highest BMI groups. The prevalence of CD (1.76% vs. 0.88% vs. 0.57%) and UC (2.52% vs. 1.20% vs. 0.97%) was higher in the older age cohort compared to early-onset and middle-age groups and more prevalent among cancer patients than controls than all age groups. Older patients had greater smoking and alcohol consumption rates compared to very early-onset and early-onset CC patients, with all p-values reported as significant.

Regarding clinical features, findings of hematochezia were noted in 8.04% of very early- and 8.67% of early-onset colon cancers and 4.18% of age >50 colon cancers. Anemia was noted in 15.69% and 15.82% of very early- and early-onset colon cancer patients and 13.80% of age >50 patients. Abdominal pain was more common in very early-onset (25.88%) and early-onset (19.86%) compared with age >50 patients (14.55%). Comparing symptoms with age-matched controls, hematochezia, weight loss, and anemia were all more common in cancer patients. Abdominal pain was less common in the very early-onset and early-onset cancer patients compared with control patients (25.88% vs. 28.01% and 19.86% vs. 25.88%). The prevalence of FAP was higher in the early-onset and middle-age cohorts compared to the older age cohort. The prevalence of JP was higher in older patients in the colon cancer cohort. Death was not significantly different across the age groups during the follow-up period. Death was more common in colon cancer patients in the very early-onset group compared with controls (0.18% vs. 0.09%), with all p-values reported reaching significance.

Rectal cancer

A total of 17,473 patients with rectal cancer were identified by claims for ICD codes and stratified by age groups. The demographics, comorbidities, and clinical features of all patients with rectal cancer are described in Table 3. The majority of patients diagnosed with rectal cancer were non-familial (96.04%) and most were over the age of 50 (75.40%). About 6.89% of patients were diagnosed prior to age 40 years (very early onset), 17.70% of patients were diagnosed between 40 and 50 years (early onset), and 75.40% were diagnosed after age 50 years. There were no significant gender differences noted across the age groups. Young and middle-age rectal patients had a lower CRC than older rectal patients (CCI >4; 2.16% vs. 3.70% vs. 8.65%; P<0.0001). The majority of the patients with rectal cancer were in the overweight and obese class, with no significant differences across the age groups or in comparison to the control group. The prevalence of CD and UC was higher in the very early-onset cohort followed by the early-onset and age >50 cohorts in that order. Findings of hematochezia (11.28% vs. 11.83% vs. 6.18%), anemia (10.2% vs. 10.6% vs. 8.80%), and weight loss (3.57% vs. 3.16% vs. 2.48%) were more common in very early- and early-onset groups than the age >50 patients. Abdominal pain (19.1% vs. 15.1% vs. 9.68%) and family history of GI malignancy (5.1% vs. 5.45% vs. 4.43%) were the most common early-onset group followed by the very early-onset and age >50 groups. Older patients had an increase in smoking (19.9% vs. 14.4% vs. 15.5%) and alcohol consumption rates (2.99% vs. 2.16% vs. 2.00%) compared to very early-onset and early-onset rectal cancer patients.

**Table 3:** Rectal cancer patients by age with controls ‡ estimated during the 12‐month baseline period

Patient characteristics	Cancer age ≤40	Controls age <40	Age 40-50	Control age 40-50	Cancer age ≥50	Controls age >50	Age ≤40 vs. ≥50	Age 40-50 vs. ≥50
	n=1,206 (6.90%)	179,828	n=3,102 (17.75%)	145,512	n=13,165 (75.34%)	n=326,697	p-value	p-value
Age (mean (SD))	34.63 (5.11)	31.45 (6.23)	45.84 (2.45)	45.25 (2.54)	57.49 (4.26)	57.02 (4.22)	<0.0001	<0.0001
Age group (n (%))							<0.0001	<0.0001
18-30	238 (19.73)	72,064 (40.07)	0	0	0	0		
30-40	968 (80.27)	107,764 (59.93)	0	0	0	0		
40-50	0	0	3,102 (100.00)	142,512 (100.00)	0	0		
50+	0	0	0	0	13,165 (100.00)	326,697 (100.00)		
Gender (n (%))							0.9376	0.4729
Male	615 (51.00)	83,743 (46.57)	1,556 (50.16)	69,755 (48.95)	6,698 (50.88)	153,545 (47.00)		
Female	591 (49.00)	96,085 (53.43)	1,546 (49.84)	72,757 (51.05)	6,467 (49.12)	173,152 (53.00)		
Year (n (%))							<0.0001	0.008
2015	401 (33.25)	55,796 (31.03)	1,148 (37.01)	44,700 (31.37)	5,108 (38.80)	104,435 (31.97)		
2016	441 (36.57)	62,922 (34.99)	1,136 (36.62)	49,379 (34.65)	4,930 (37.45)	112,873 (43.55)		
2017	364 (30.18)	61,110 (33.98)	818 (26.37)	48,433 (33.99)	3,127 (23.75)	109,389 (33.48)		
Region of residence (n (%))							0.053	0.1369
Northeast	205 (17.00)	31,820 (17.69)	533 (17.18)	25,132 (17.64)	2,464 (18.72)	62,338 (39.08)		
Northcentral	216 (17.91)	31,491 (17.51)	614 (19.79)	24,061 (16.88)	2,699 (20.50)	58,772 (17.99)		
South	600 (49.75)	87,194 (48.49)	1,496 (48.23)	72,117 (50.60)	6,196 (47.06)	159,386 (48.79)		
West	179 (14.84)	28,947 (16.10)	444 (14.31)	20,872 (14.65)	1,756 (13.34)	45,287 (13.86)		
Unknown	6 (0.50)	376 (0.21)	15 (0.48)	330 (0.23)	50 (0.38)	914 (0.28)		
Patient risk factors ‡								
Charlson comorbidity index (Mean (SD))	1.57 (0.76)	0.37 (0.67)	1.65 (0.87)	0.61 (0.90)	1.89 (1.12)	1.02 (1.23)		
Charlson comorbidity index (n (%))							<0.0001	<0.0001
0	0	128,096 (71.23)	0	83,186 (58.37)	0	141,664 (43.36)	<0.0001	<0.0001
1	675 (55.97)	40,608 (22.58)	1,664 (53.64)	40,458 (28.39)	6,116 (46.46)	98,533 (30.16)		
2	405 (33.58)	8,522 (4.74)	1,032 (33.27)	12,999 (9.12)	4,180 (31.75)	49,041 (15.01)		
3	99 (8.21)	1,903 (1.06)	291 (9.38)	3,961 (2.78)	1,737 (13.19)	21,860 (6.69)		
4+	27 (2.24)	699 (0.39)	115 (3.71)	1,908 (1.34)	1,132 (8.60)	15,599 (4.77)		
BMI (n (%))								
≤25	29 (2.40)	3,011 (1.67)	76 (2.45)	1,645 (1.13)	360 (2.73)	4,795 (1.46)	0.946	0.6015
25-30	44 (3.65)	4,265 (2.37)	118 (3.80)	4,362 (3.00)	467 (3.55)	11,972 (3.66)		
30-35	31 (2.57)	5,437 (3.02)	107 (3.45)	5,617 (3.86)	390 (2.96)	13,197 (4.04)		
35-40	15 (1.24)	4,174 (2.32)	41 (1.32)	3,979 (2.73)	159 (1.13)	7,903 (2.42)		
40+	27 (2.24)	11,936 (6.64)	72 (2.32)	9,079 (6.24)	307 (2.33)	14,462 (4.43)		
Family history of GI malignancies (n (%))	62 (5.14)	2,253 (1.25)	169 (5.45)	4,218 (2.96)	583 (4.43)	12,087 (3.70)	0.2527	0.015
Personal history of other non-GI malignancies (n (%))	136 (11.28)	664 (0.37)	406 (13.09)	1,625 (1.14)	1,929 (14.65)	8,513 (2.61)	0.0014	0.0254
Crohn's colitis (n (%))	26 (2.16)	1,877 (1.04)	24 (0.77)	968 (0.68)	62 (0.47)	1,777 (0.54)	<0.0001	0.0365
Ulcerative colitis (n (%))	37 (3.07)	1,350 (0.75)	39 (1.26)	878 (0.62)	109 (0.83)	2,161 (0.66)	<0.0001	0.0235
Peutz-Jeghers (n (%))	1 (0.08)	26 (0.01)	0	20 (0.01)	1 (0.01)	32 (0.01)	0.0338	0.6274
Juvenile polyposis (n (%))	38 (3.15)	284 (0.16)	91 (2.93)	529 (0.37)	424 (3.22)	4,028 (1.23)	0.8954	0.4114
Familial adenomatous polyposis (n (%))	12 (1.00)	221 (0.12)	41 (1.32)	493 (0.35)	98 (0.74)	1,542 (0.47)	0.3392	0.0017
Smoking (n (%))	174 (14.43)	26,554 (14.77)	482 (15.54)	20,638 (14.48)	2,629 (19.97)	54,890 (16.80)	<0.0001	<0.0001
Alcohol (n (%))	26 (2.16)	7,857 (4.37)	62 (2.00)	6,182 (4.34)	394 (2.99)	15,135 (4.63)	0.0986	0.0025
Statins (n (%))	7 (0.58)	1,342 (0.75)	96 (3.09)	5,025 (3.53)	922 (7.00)	27,354 (8.37)	<0.0001	<0.0001
Patient index symptoms								
Hematochezia (n (%))	136 (11.28)	2,630 (1.46)	367 (11.83)	2,063 (1.45)	813 (6.18)	4,879 (1.49)	<0.0001	<0.0001
Anemia (n (%))	123 (10.20)	10,833 (6.02)	329 (10.61)	10,561 (7.41)	1,159 (8.80)	26,817 (8.21)	0.1037	0.0017
Dysphagia (n (%))	9 (0.75)	2,046 (1.14)	34 (1.10)	2,420 (1.70)	144 (1.09)	6,767 (2.07)	0.2604	0.9913
Abdominal pain (n (%))	231 (19.15)	50,361 (28.01)	469 (15.12)	36,880 (25.88)	1,275 (9.68)	74,833 (22.91)	<0.0001	<0.0001
Weight loss (n (%))	43 (3.57)	1,886 (1.05)	98 (3.16)	1,480 (1.04)	326 (2.48)	5,517 (1.69)	0.0221	0.0317
GI bleeding (n (%))	63 (5.22)	1,161 (0.65)	132 (4.26)	1,210 (0.85)	416 (3.16)	4,150 (1.27)	0.2527	0.015
Vitamin D deficiency (n (%))	52 (4.31)	13,330 (7.41)	177 (5.71)	14,531 (10.20)	759 (5.77)	34,992 (10.71)	0.0363	0.8985
Death (n (%))	1 (0.08)	156 (0.09)	6 (0.19)	200 (0.14)	24 (0.18)	1,224 (0.37)	0.428	0.8967

The demographics, comorbidities, and presentation of all patients with rectal after excluding patients with familial gastrointestinal diseases are described in Table 4. There were similar demographic trends across age groups in rectal patients without familial gastrointestinal diseases compared to all rectal patients described in the previous paragraph. Trends were noticed across gender, CCI, BMI, various clinical findings, smoking, and alcohol as described previously across all rectal cancer patients. Limited analysis between age-matched non-cancer-affected controls and cancer patients revealed few novel features between these groups and was limited to comparison with control with CLD so it served primarily as a surrogate baseline demographic.

**Table 4:** Non-familial patients with rectal cancer ‡ estimated during the 12‐month baseline period

Patient characteristics	Age ≤40	Age 40-50	Age ≥50	Age ≤40 vs. ≥50	Age 40-50 vs. ≥ 50
	n=1,157 (6.89%)	n=2,971 (17.70%)	n=12,653 (75.40%)	p-value	p-value
Age (mean (SD))	1.41 (0.76)	45.84 (2.45)	57.49 (4.26)	<0.0001	<0.0001
Gender (n (%))				0.9453	0.636
Male	589 (50.91)	1,495 (50.32)	6,428 (50.80)		
Female	568 (49.09)	1,476 (49.68)	6,225 (49.20)		
Comorbidity profile ‡					
Charlson comorbidity index (mean (SD))	1.58 (0.76)	1.65 (0.87)	1.90 (1.12)	<0.0001	<0.0001
BMI				0.9681	0.2648
≤25	28 (20.29)	68 (17.35)	345 (21.81)		
25-30	42 (30.43)	110 (28.06)	440 (27.81)		
30-35	30 (21.74)	105 (26.79)	365 (23.07)		
35-40	13 (9.42)	40 (10.20)	145 (9.17)		
40+	25 (18.12)	69 (17.60)	287 (18.14)		
Crohn's colitis (n (%))	26 (2.25)	23 (0.77)	55 (0.43)	<0.0001	0.0181
Ulcerative colitis (n (%))	35 (3.03)	38 (1.28)	90 (0.71)	<0.0001	0.002
Personal history of other non-GI malignancies (n (%))	128 (11.06)	391 (13.16)	1,867 (14.76)	0.0006	0.0261
Smoking (n (%))	168 (14.52)	468 (15.75)	2,529 (19.99)	<0.0001	<0.0001
Alcohol (n (%))	23 (1.99)	61 (2.05)	383 (3.03)	0.0452	0.004
Statins (n (%))	6 (0.52)	91 (3.06)	876 (6.92)	<0.0001	<0.0001
Clinical features					
Hematochezia (n (%))	124 (10.72)	336 (11.31)	734 (5.80)	<0.0001	<0.0001
Anemia (n (%))	117 (10.11)	302 (10.16)	1,101 (8.70)	0.1053	0.012
Dysphagia (n (%))	9 (0.78)	32 (1.08)	135 (1.07)	0.3542	0.9614
Abdominal pain (n (%))	214 (18.50)	432 (14.54)	1,201 (9.49)	<0.0001	<0.0001
Weight loss (n (%))	37 (3.20)	97 (3.26)	314 (2.48)	0.1384	0.0164
GI bleeding (n (%))	56 (4.84)	118 (3.97)	386 (3.05)	0.0009	0.0106
Vitamin D deficiency (n (%))	45 (3.89)	165 (5.55)	706 (5.58)	0.0152	0.9556
Death (n (%))	1 (0.09)	6 (0.20)	24 (0.19)	0.4291	0.8906

## Discussion

The data presented in this analysis further emphasizes the increasing prevalence of CRC in young patients over the last 30 years [2]. The goal of this retrospective claims-based analysis was to expand on the previous series by using the large MSCC insurance claims database to examine age-based cohorts for both colon and rectal cancer within the United States for additional trends, risk factors, and clinical features. Differences in morbidity and treatment for rectal cancer made this differentiation significant and have not been consistently reported in the previous series. Considering the case series had shown a significant number of cases in patients age <40, this group was analyzed separately in a group termed “very early onset,” to help differentiate any features in this group currently not recommended for routine screening [3].

Among colon cancer patients in our cohort, we found 24.03% presented at age 18 to 50, a slight increase in prevalence in this population compared with previous SEER database analysis showing this group made up 21.2% of total CRC cases up to 2013 [15]. About 7.58% of patients were diagnosed at age 18 to 40 years old, and in this group <10% reported a family history of GI malignancy. Subgroup analysis excluding familial syndromes and patients with family history did not show an appreciable change in these age-based percentages, indicating that the vast majority of early- and very early-onset cases were sporadic in nature. A higher proportion of females was noted in the two early-onset colon cancer groups compared with the age >50 group, although it should be noted that our control group had a similarly increased proportion of females in the younger groups as well. This suggests that female sex is a risk factor for early-onset colon cancer, although comparison with a more standardized control group is needed to solidify this correlation. Recent studies have demonstrated an association with sex hormones and genetic variants in hormone metabolic pathways that contributed to susceptibility to CRC, which could explain the difference in the incidence between the sexes [16]. Gender was a less significant risk factor for rectal cancer, which showed a slight male predominance.

Comorbidity analysis in the early- and very early-onset colon cancer groups revealed a significant proportion with at least two comorbidities by the CCI, with the highest percentage having three or four comorbidities. Obesity has been implicated in previous series as a likely risk factor for CRC, and in our series, the majority of both colon and rectal cancer patients in all groups were in the overweight, obese, or morbidly obese weight categories. Of note, morbidly obese patients with BMI >40 were highly represented in the colon cancer population across all age groups, representing 24.94%, 25.75%, and 21.34% of colon cancer cases in age ≤40, 40-50, and ≥50 groups, respectively. Interestingly, the proportion of patients in the obese groups was similar between the rectal cancer age groups, which is in contrast to the previous series which implicated obesity as a rectal cancer risk factor [17]. The nature of claims analysis for obesity makes tracking these trends cumbersome, as there are multiple ICD codes used by different providers that may not have been captured in this analysis which could make correlations less clear. Comparison with controls was also not helpful in this case, as our control population was enriched with a significant proportion of cases with NAFLD and a higher proportion of obesity than the cancer groups in most age groups.

Regarding clinical features in colon cancer patients, a larger proportion of age 18 to 40 and age 40 to 50 patients presented with abdominal pain compared with the age 50 to 65 group (25% vs. 19% and 14%, respectively). While abdominal pain is common in control groups and a frequent feature of an ultimately benign work-up by gastroenterologists, this suggests a high index of suspicion is warranted even in younger patients when tracking this symptom over time. Weight loss and hematochezia were also significantly more common in the younger age groups compared with older cancer patients. Age 18-40 and 40-50 patients with rectal cancer were more likely to present with hematochezia than age 50-65 patients. Previous series have noted that patients with early-onset (age <50) CRC experience symptoms for a longer duration of time and a longer delay in diagnosis compared with late-onset CRC [18,19], likely a result of lower index suspicion with younger patients in the past. Expanding the understanding of this increasing incidence and maintaining a high index of suspicion are crucial aspects for clinicians in identifying early-onset CRC cases.

Multiple factors have been hypothesized to account for this proportional increase in early-onset CRC, including obesity, a non-Mediterranean Western diet, a sedentary lifestyle, low fiber intake, and dysbiosis [20]. Some associated genetic loci have been accounted for, including differential expression of genes in early-onset sporadic CRC including TNFR1, EIF4E, CLC, and IFNAR1 [21,22]. There is a possible relationship between microsatellite instability of tumors in younger patients with CRC [8]. Nonetheless, there remains a lack of consensus on whether early-onset CRC has a distinct molecular/immunologic entity from CRC in older patients [8,23-25]. Some data already support the possible benefit of screening younger adults for CRC based on microstimulation analyses [26]. In the last three years, the American Cancer Society and the American College of Gastroenterology, as well as the US Protective Task Force, have issued recommendations in support of earlier screening at age 45 [27].

The main strengths of this analysis include the very large cohort analyzed, including >15,000 CRC cases in patients age <50, and the novel analysis of very early-onset cases in patients age <40 for both colon and rectal cancers. This is larger in size than the recent MSCC analysis focusing mainly on metabolic syndrome and obesity [28] and larger than the recent VA series which included 12,229 total cases with 97% men [29]. Previous analyses all focused on adults age 18-49 as the early-onset colon cancer group; thus, focusing on the age group 18-40 represents a large cohort not previously cited in other series. Claims data allowed for analysis of clinical complaints and symptoms identified, which has also not been a focus of recent large series in younger age groups. The analysis does have weaknesses, including that Lynch syndrome was not easily extracted from the claims database and was not included in this analysis, which excludes an important cause of early-onset genetic cancer syndromes. The analysis was also weakened by the lack of an easily comparable control group from which reliable odds ratios could be calculated, making the generalizability of associations less reliable. We used ICD codes to analyze patients' symptoms, but providers may not be assigning diagnosis codes for mild or even moderate symptoms, so some symptoms are likely not being accounted for. Furthermore, the ICD code definitions themselves are non-validated which introduces a confounding factor into our study. Recent series mentioned in this paper previously have already highlighted some of these risk factors compared with controls, particularly with respect to obesity and diet, making some of these features noted less novel. This analysis does emphasize that further comparison of sex differences and clinical symptoms in these populations compared with controls is warranted.

## Conclusions

CRC in patients age <50 represents a growing proportion of cases. Current screening guidelines are adapting to capture more of these patients; however, identification of additional risk factors is needed to help identify those at the highest risk that may warrant earlier screening. Our cohort contributes to the data demonstrating a significant proportion (~25%) of colon and rectal cancer patients being diagnosed under the age of 50 years, with 8% presenting younger than age 40. We found notable differences among genders across various age groups, including a higher proportion of female colon cancer cases among patients under the age of 40. Obesity and high BMI levels remain important risk factors across age groups, with a high proportion of these patients in the highest BMI category for colon cancer. Clinical differences were noted among younger patients, including more colon and rectal cancer cases reporting abdominal pain, hematochezia, and weight loss compared with cases older than 50. Many patients reported a family history in this cohort, but the majority of cases were sporadic, with a proportion of cases not changing by age group after excluding familial cases, highlighting the importance of a high index of suspicion even outside of a strong family history. Further analysis of risk features in this young cohort can help improve earlier clinical detection and limit the time to diagnosis in patients who may be at risk.
